# An Observatory to monitor range extension of the Mediterranean monk seal based on its eDNA traces: collecting data and delivering results in the “Open Science” era

**DOI:** 10.3897/BDJ.12.e120201

**Published:** 2024-06-05

**Authors:** Elena Valsecchi, Alessandro Gabbiadini

**Affiliations:** 1 University of Milano-Bicocca, Milano, Italy University of Milano-Bicocca Milano Italy; 2 MaRHE Center, Magoodhoo, Maldives MaRHE Center Magoodhoo Maldives

**Keywords:** barcoding, qPCR, single-species assay, citizen science, molecular monitoring, environmental DNA

## Abstract

The monk seal is the most endangered pinniped in the world and the only one found in the Mediterranean, where its distribution and abundance have suffered a drastic decline in the last few decades. Data on its status are scattered due to both its rarity and evasiveness and records are biased towards occasional, mostly coastal encounters. Nowadays, molecular techniques allow us to detect and quantify minute amounts of DNA traces released into the environment (eDNA) by any organism. A species-specific molecular assay is now available for detecting the recent presence of the monk seal in the water column through the analysis of sea-water samples collected from the sea surface. The project “Spot the Monk” uses this non-invasive detection tool to monitor monk seal occurrence in Mediterranean waters by means of eDNA analysis. The simplicity in the acquisition of samples together with the need to collect samples in multiple points simultaneously made the project well suited to the involvement of the general public. Up to today, about 350 samples have been collected and analysed in the central-western Mediterranean by researchers and a multifarious range of citizen scientists - from recreational sailing organisations, both amateur and competitive sportsmen, to fishermen. This work announces the launch of an open-source Observatory (https://www.spot-the-monk-observatory.com/) where the project outcomes are publicly accessible as soon as they are produced. Embracing the principles of Open Science, we believe that such an approach can contribute to filling the knowledge gap about the distribution of this charismatic species in our seas, providing, at the same time, a proof of concept on how data collected by a variety of actors can be returned to the scientific and non-scientific communities in an innovative format for immediate consultation.

## Science in a changing world

Since the beginning of the new millennium, each field of Science has undergone exponential growth, following the surge in the development of new technologies, an increasingly larger scientific community involved in research and the help and exploitation of artificial intelligence (AI), leading to the beginning of the era of big data. In the field of molecular ecology, the revolutionary breakthrough has been acquired through technological competence to "read" the DNA that each living organism releases into the environment in which it lives (eDNA). These techniques are becoming so sensitive that they allow for the identification of infinitesimal traces of a specific target species’ eDNA, even if this is rare and difficult to observe. In parallel with technological development, there is a emergent awareness that there is an entire world to be sampled, sequenced and analysed so that it may be better understood and safeguarded. Not only, the general public can play a key role in this process (Citizen Science). Definitely a new era for Science.

## The Mediterranean monk seal conservation profile

The Mediterranean Monk Seal, *Monachusmonachus* (Hermann 1779), is the only pinniped endemic to the Mediterranean Sea. This species is currently listed as vulnerable according to The International Union for Conservation of Nature (IUCN) Red List (Karamanlidis et al. 2023) and, although showing in the last decade weak signs of population expansion, it is still considered one of the most threatened pinniped species in the world (Karamanlidis 2024). Once widely spread in the Mediterranean, the Monk Seal was eradicated from most of its former range well before the Second World War (Johnson 2004). This species was exploited for subsistence needs, commercial harvest for skin and oil and persecuted as a competitor for fishery resources or because it caused actual and perceived damage to fishing gear. With the exception of the Aegean Sea, the core area of distribution for the residual population in the Mediterranean, direct observations in adjacent areas are still relatively scarce, making it difficult to understand species distribution, home range, habitat use and seasonal movement.

## Project description: from *Spot the Monk* to its Observatory

*Spot the Monk*, (StM,) is an initiative born in 2019, after the molecular identification system for the Mediterranean monk seal was developed and validated. Since then, StM has been continuously expanding and developing. Not only were several scientific or Citizen-Science associations involved in the sampling, but also some Marine Protected Areas and parallel scientific projects flanked the venture. A series of progressive BioBlitzes is currently underway (sampling every 6 weeks, throughout the year). To cope with the drastic increase in data (which will more than double in 2024), it was necessary to create a platform where all results were simultaneously viewable.

The project is constantly developing and growing. The next steps concern the implementation of the molecular methodology (e.g. updating the assay with more sensitive approaches, for example, ddPCR), the integration of the Observatory in global data aggregators, such as the European Marine Observation and Data Network (EMODnet) and the Open Science Framework (https://osf.io/) and the enlargement of the sampling area (e.g. including the North African coast).

## Data displayed and their relevance

**Figure 1.**
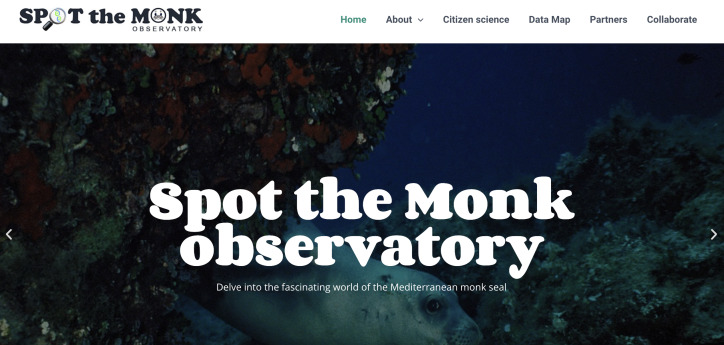
Home page of the Spot the Monk Observatory website.

The Observatory (Fig. 1) is conceived as a repository of the results of the screening carried out on eDNA samples (currently around 450) collected since 2018 in the Mediterranean, displaying where samples tested positive for the presence of traces of monk-seal eDNA. The primer, MmoMV2R, used to target a species-specific region of the mitochondrial 12S-rDNA, is deposited in the BOLD System database (https://www.boldsystems.org/index.php/Public_Primer_PrimerSearch). We used it in a qPCR assay, whose efficiency has been validated and the protocol for its use is described in Valsecchi et al. (2022) and Valsecchi et al. (2023), respectively.

Although the fate and persistence of eDNA in the aquatic environment is still not fully understood, since it depends also on the source organism (dimensions, habits and habitats) and the biological material from where it originates (e.g. sloughed skin, faeces etc.), as well as being affected by many biotic (e.g. entering the food web) and abiotic (e.g. UV, currents) factors (Barnes 2014), yet eDNA is emerging as a powerful tool for community assessment and monitoring and holds the promise of revolutionising the study of marine biodiversity (Takahashi et al. 2023). In our specific case, positive detections can still be informational in acquiring indicators regarding the marine districts visited by this elusive species.

Trace detectability is tightly associated with signal dispersal, as biological traces move with currents, tend to disperse and, thus, become diluted along with the time since they were shed into the water column. For this reason, the concentration of the signal can give insights into the freshness of the trace. The concentration is, in turn, proportional to the number of experimental replicates (out of nine according to the Spot the Monk protocol, Valsecchi et al. 2022 and Valsecchi et al. 2023) returning a positive detection. In order to simplify the presentation of outcomes, in the Observatory, all the samples returning at least one positive replicate are reported as positive.

## Researchers, conservation stakeholders and citizen scientists linked together

The Observatory is intended as a forum where professional and amateur expertise meets, carries forward data and information that provide a feedback loop for all stakeholders to advance and develop a collaborative approach to explore new pathways, in alignment with the new EU research specification (https://research-and-innovation.ec.europa.eu/strategy_en).

## The Observatory’s features

1. Real-Time Data Display: Data are displayed as soon as they are analysed. Once samples are processed, the results are seamlessly integrated, offering an up-to-date overview of the evidence of monk seal distribution in the Mediterranean.
2. Progressive Data Display: the gradual and cumulative data display allows one to view all processed data (published and still unpublished) at once, permitting one to gather a higher and higher resolution of the species' distribution, seasonal movements and home range as time passes.
3. Data-Filtering Option: data consultation is facilitated by the possibility of filtering the data display by year and outcome (positive or negative) of the molecular screening. Further filtering options will be implemented in the future.
4. Simple Language: we opted for a straightforward and attractive language in order to entertain the widest range of users, including the younger generations, fundamental actors in the future protection of the species.
5. Accessible to All: The Spot the Monk Observatory is designed for everyone. Whether you are an experienced scientist or a curious enthusiast, the platform welcomes everybody to explore the Spot-the-Monk results in real time. The data are presented in an easily understandable format, bridging the gap between scientific research and public engagement.
6. Quicker Return of Citizens' Data Results: the possibility of relying on Citizen-Science contribution is one of the great advantages of environmental DNA studies - the sample collection phase is very simple. However, effective Citizen-Science campaigns require a prompt return of the results obtained from the volunteers’ contribution, in order to both reward them for their efforts and to encourage them to persevere in similar Citizen-Science programmes in the future. This has not always been possible in molecular studies as this process is severely slowed by the prolonged time needed for data analysis and result publication. This can be overcome by the progressive data display, as shown by the Observatory.
7. Increased Visibility of Collaborators: a website, such as the Observatory, provides a more appropriate and rewarding form of acknowledgement for non-scientific collaborators (as opposed to having their names listed in the Acknowledgements section of a scientific paper).
8. Educational Insights: the Observatory was implemented with an educational dimension, explaining the molecular techniques employed, the significance of environmental DNA analysis and the critical role each person plays in the conservation of this vulnerable species.
9. The web-based information technology infrastructure is founded upon completely open source software (Wordpress Content mangement system; OpenStreetMap API). This approach allowed us to develop points 1 to 3.
10. The Observatory fully supports the approach and release of FAIR (Findable, Accessible, Interoperable and Reusable) data (Wilkinson 2016).
